# Comparison Between Varicocelectomy and Varicocele Sclerotherapy: Results of a Single-Center Observational Study

**DOI:** 10.3390/life14111368

**Published:** 2024-10-24

**Authors:** Rossella Cannarella, Vittorio Cannarella, Rosario Randazzo, Andrea Crafa, Michele Compagnone, Laura M. Mongioì, Rosita A. Condorelli, Vincenzo Bagnara, Sandro La Vignera, Aldo E. Calogero

**Affiliations:** 1 Department of Clinical and Experimental Medicine, University of Catania, Via S. Sofia 78, 95123 Catania, Italy; cannarella.vittorio@gmail.com (V.C.); dott.randazzorosario@gmail.com (R.R.); crafa.andrea@outlook.it (A.C.); michele.compagnone22@tiscali.it (M.C.); lauramongioi@hotmail.it (L.M.M.); rosita.condorelli@unict.it (R.A.C.); sandrolavignera@unict.it (S.L.V.); acaloger@unict.it (A.E.C.); 2 Glickman Urological and Kidney Institute, Cleveland Clinic, Cleveland, OH 44195, USA; 3 Department of Pediatric Surgery, Policlinico “G.B.Morgagni”, 95125 Catania, Italy; vincenzobagnara@gmail.com

**Keywords:** varicocele, sclerotherapy, varicocelectomy, sperm parameters, bio-functional

## Abstract

Affecting up to 15% of men worldwide, varicocele has been recognized as a cause of infertility, and its repair is associated with an improvement in conventional and bio-functional sperm parameters. Various surgical and radiological techniques exist for varicocele repair. However, it is unclear which technique is associated with greater clinical efficacy. This retrospective, single-center study aimed to compare the effectiveness of surgical treatment (Ivanissevich technique) versus radiological treatment (sclerotherapy) in a cohort of 94 patients with varicocele. After varicocele repair, a significant increase in sperm concentration was observed only in the group of patients treated with sclerotherapy. A significant reduction in the percentage of patients with oligozoospermia was found in the group of patients treated surgically. Patients undergoing surgical varicocelectomy had increased serum luteinizing hormone (LH) levels, decreased spermatid concentration, and increased percentage of spermatozoa in late apoptosis, probably as a result of surgical traumatism. In conclusion, the results of this study did not show a clear benefit of one technique over the other and confirm the findings of the current literature. However, it remains one of the few on the topic that also considers sperm bio-functional parameters among its outcomes and opens the research up to new considerations on the bio-functional sperm parameters.

## 1. Introduction

Varicocele is an abnormal dilatation of the pampiniform plexus, the venous circle deputed for draining deoxygenated blood from the scrotum [1]. It is described as one of the main causes of secondary male infertility [2]. For many years, varicocele was thought to occur prevalently on the left side, but nowadays data collected by venography, thermography, and ultrasound suggest bilateral disease in over 80% of cases [3]. Epidemiological data indicate that varicocele affects 15% of the world’s male population, in particular, 25–35% of patients with primary infertility, and 50–80% with secondary infertility [4,5,6,7,8]. It also appears more prevalent in patients with venous insufficiency [9] or with a positive family history [10], suggesting a relationship between varicocele and venous disease and genetics.

The relationship between varicocele and infertility is controversial. Some studies indicate an increased likelihood of pregnancy after varicocelectomy [11], while others report pregnancies even in patients with untreated varicocele [12].

It is known that venous dilatation causes blood stasis with an increased scrotal temperature [13,14]. In turn, heat damages the testis and its proteins.

Moreover, several lines of evidence have demonstrated the positive impact that varicocele repair has on conventional sperm parameters and sperm DNA fragmentation (SDF). In particular, a recent systematic review and meta-analysis including 16 controlled studies compared conventional sperm parameters post-varicocele repair with those of patients with untreated varicocele, and showed a significant improvement in sperm concentration, total sperm count, progressive motility, and percentage of spermatozoa with normal morphology [15]. The same group of authors reported the same results using a pre–post approach, consisting of comparing post-treatment conventional sperm parameters with pre-treatment ones [16]. Interestingly, varicocele repair also resulted in a reduction of SDF rate from baseline values, as well as of seminal malondialdehyde (MDA), a marker of oxidative stress, in a meta-analysis of 29 studies [17].

Treatment of varicocele is based on surgical and radiological techniques. Surgical procedures include sub-inguinal varicocelectomy, retroperitoneal varicocelectomy (also known as Palomo, Ivassevich technique), or inguinal varicocelectomy. These techniques surgically isolate the dilated spermatic vein, which is reached using a cutaneous approach, the level of which differs depending on the specific procedure. Radiological techniques include embolization, the selective closure of dilated vessels using a balloon or coil, and sclerotherapy, in which the spermatic vein is ligated after being treated with a sclerosing agent that damages its intimal tunica and promotes its narrowing. The current literature does not provide sufficient insights to establish whether surgical treatment is superior to radiological treatment. In particular, the latest Cochrane systematic review on this topic, which collects all available evidence, does not conclude in favor of one procedure over the others because of the uncertain evidence [18]. With these premises, this retrospective study aimed to compare the efficacy of surgical (Ivanissevich technique) versus radiological (sclerotherapy) treatment on testicular function in a cohort of 94 patients with varicocele. The outcomes of the study were conventional and bio-functional sperm parameters, serum hormone levels, and testicular volume.

## 2. Patients and Methods

### 2.1. Patients

This observational study includes 94 patients [median age: 29.0 (23.0–36.0)] with varicocele (III–V degree according to the Sarteschi classification) [19]. The sample size was calculated for continuous outcomes using the PASS Software, version 24.0.2 (PASS 13, Hintze, (2014); PASS 13, NCSS, LLC, Kaysville, UT, USA; www.ncss.com) considering a power of 80% and accepting an alpha error of 0.05. All patients were referred to the Division of Endocrinology, Metabolic Diseases and Nutrition, University Teaching Hospital Policlinico “G. Rodolico—San Marco”, University of Catania, Catania, Italy.

We excluded patients whose information about sperm parameters and ultrasound features before surgery was missing. We also excluded patients with follicle-stimulating hormone (FSH) serum levels > 8 mIU/mL, primary testicular diseases other than varicocele, male accessory gland infections, central hypogonadism and other endocrine diseases, systemic diseases, chronic exposition to occupational toxicants, intake of spermiotoxic drugs, cigarette smoking, alcohol, and drug abuse, and consumption of nutraceuticals with antioxidant effects.

Testicular volume and varicocele degree were evaluated by testicular ultrasound end echo-color Doppler before and after varicocele repair. Varicocele was classified according to the Sarteschi classification [19]. The same experienced physicians fully trained in scrotal ultrasound (M.C.) performed the ultrasound evaluation before and after varicocele repair. We excluded patients with varicocele degree ≤ II.

Hormone levels, testicular volumes, and conventional and bio-functional sperm parameters were evaluated before and after varicocele repair (5.1 ± 0.57 months, range 3–13 months).

### 2.2. Serum Hormone Measurements

Blood sampling was performed at 8.00–9.00 a.m., after at least 8 h of sleep. The serum concentrations of FSH, luteinizing hormone (LH), and total testosterone (TT) were measured using a chemiluminescent microparticle immunoassay (ARCHITECT System, Abbott, Longford, Ireland). Normal ranges were FSH = 2.0–12.0 mIU/mL, LH = 1.6–9.0 mIU/mL, and TT = 3.5–8.0 ng/mL (the grey zone for TT was defined as for values 2.3–3.4 ng/mL).

### 2.3. Semen Analysis and Bio-Functional Sperm Parameter Evaluation

Each patient was asked to collect semen samples by masturbation after 3–4 days of sexual abstinence if there had been no fever, genital tract infection, severe trauma, and/or surgery had occurred in the three months preceding examinations. Semen analysis was conducted according to the WHO 2010 criteria [20]. We evaluated the following bio-functional sperm parameters by flow cytometry: alive and apoptotic spermatozoa, spermatozoa with low mitochondrial membrane potential (MMP), spermatozoa with low degree of chromatin compactness, and increased SDF rate.

Flow cytometry analysis was performed using the flow cytometer CytoFLEX (Beckman Coulter Life Science, Milan, Italy). CytoFLEX has equipped with two solid-state lasers at 488 and 638 nm and with seven fluorescence channels: 525/40 BP, 585/42 BP, 610/20 BP, 690/50 BP, 780/60 BP for excitation at 488 nm and 660/10 BP, 712/25 BP, 780/60 BP for excitation at 638 nm. Data were analyzed by the software CytExpert 1.2. Bio-functional sperm parameters were evaluated as described elsewhere [21].

### 2.4. Varicocele Repair

Varicocelectomy was performed following the Ivanissevich technique, after the incision of the external oblique fascia above the inguinal ring in order to introduce the spermatic cord into the operative field [22].

The right common femoral vein or basilic vein was identified ultrasonographically and used as percutaneous access for sclerotherapy, as described by Mongioì and colleagues [21]. Briefly, a 21-gauge micropuncture set (Prelude EASE, Merit medical system, South Jordan, UT, USA) was used to position an introducer. A 0.035 hydrophilic guidewire (Terumo Corp., Tokyo, Japan) was used to reach the distal portion of the left spermatic vein, which was catheterized with a 5 Fr C2 or C1 Cobra-shaped angiographic diagnostic catheter (Terumo corp., Tokyo, Japan). After performing a diagnostic phlebography to study the venous circulation in order to look for collateral branches in the venous gonadal drainage [23], a balloon (Rival, Bard Peripheral Vascular, Tempe, AZ, USA; size between 4 and 8 mm depending on the diameter of the vein) was introduced distal to the upper margin of the left iliac bone and inflated to interrupt the retrograde flow. A second phlebography was then carried out and, if systemic collaterals were observed, a mixture of contrast agent and sclerosant was injected, checking and stopping the injection once the proximal part of collateral was visualized. The occluding balloon was then deflated and a control phlebography was performed.

### 2.5. Statistical Analysis

Data are shown as mean ± standard deviation (SD) for non-skewed variables, while non-normally distributed continuous variables are shown as median and interquartile range (IQR). The distribution of values was evaluated using the Shapiro–Wilk test. The inter-group comparison was performed using the one-way analysis of variance (ANOVA) for normal distributed variables, the ANOVA test for log-transformed data, or the Kruskal–Wallis test for non-normally distributed data. Statistical analysis was performed using MedCalc Software Ltd. (Ostend, Belgium), version 19.6-64-bit. A *p*-value less than 0.05 was considered statistically significant.

### 2.6. Ethical Approval

This study was conducted at the Division of Endocrinology, Metabolic Diseases and Nutrition of the University Teaching Hospital Policlinico “G. Rodolico”, University of Catania (Catania, Italy), in accordance with the ethical principles of the Declaration of Helsinki and its later amendments Informed consent was obtained from each patient after a full explanation of the purpose and nature of all procedures that are commonly used in clinical practice. 

## 3. Results

### 3.1. Entire Population

The general characteristics of patients at enrollment are reported in Table 1. Overall, 25 patients (26.6%) had oligo-astheno-teratozoospermia, 4 (4.3%) were normozoospermic, and the other 65 (69.1%) had one or two sperm parameter abnormalities. At least one abnormality of sperm parameters was found in 90.9% and 78% of patients in the varicocelectomy group and the sclerotherapy group, respectively. Patients treated with sclerotherapy had a lower recurrence rate than patients who underwent varicocelectomy, but the difference did not reach statistical significance. No difference was observed between the two groups in terms of complication rate.

**Figure 1:**
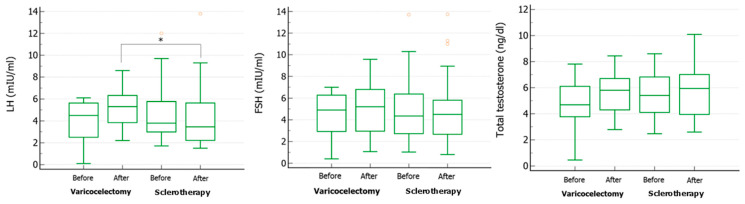
Serum levels of luteinizing hormone (LH) (**left panel**), follicle-stimulating hormone (FSH) (**middle panel**), and total testosterone (**right panel**) in patients with varicocele before and after undergoing varicocelectomy (Ivanissevich technique) or sclerotherapy. * *p* < 0.05, One-way analysis of variance (ANOVA) or Kruskal–Wallis test.

Regarding hormonal evaluation, post-treatment LH was significantly higher in the varicocelectomy group than in the sclerotherapy group (Figure 1, left panel). No difference in serum FSH (Figure 1, middle panel) and TT (Figure 1, right panel) levels was found.

**Figure 2:**
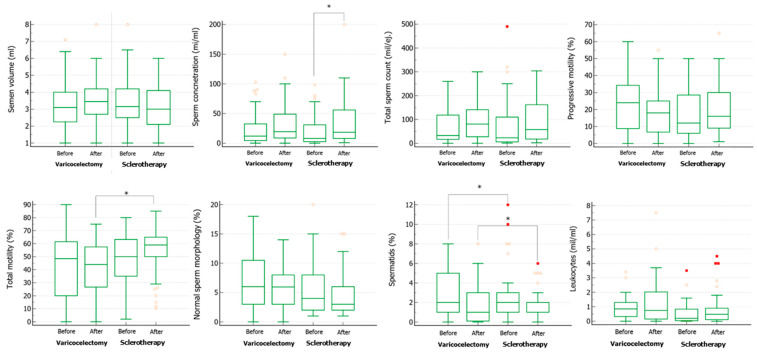
Conventional sperm parameters in patients with varicocele before and after undergoing varicocelectomy (Ivanissevich technique) or sclerotherapy. * *p* < 0.05, One-way analysis of variance (ANOVA).

Seminal volume increased in both groups after varicocele repair, but not significantly (Figure 2). Sperm concentration increased significantly only in patients who underwent sclerotherapy, while no difference was found for total sperm count, sperm progressive motility, percentage of sperm with normal morphology, and leukocyte concentration. Total motility showed a significant increase in patients undergoing sclerotherapy (Figure 2). Finally, a significant reduction in spermatid concentration was found in patients who underwent surgical varicocelectomy (Figure 2).

**Figure 3:**
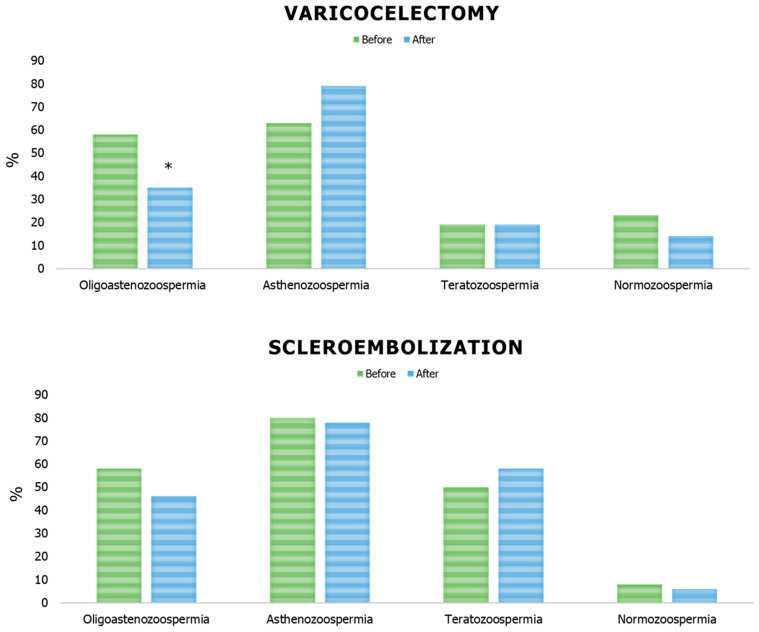
Sperm parameter abnormalities in patients with varicocele before and after undergoing varicocelectomy (Ivanissevich technique) or sclerotherapy. * *p* < 0.05, Chi-squared test.

However, considering the presence of abnormalities in conventional sperm parameters, a significant reduction in the percentage of patients with oligozoospermia was found after varicocelectomy, while no change was found in the group undergoing sclerotherapy (Figure 3).

**Figure 4:**
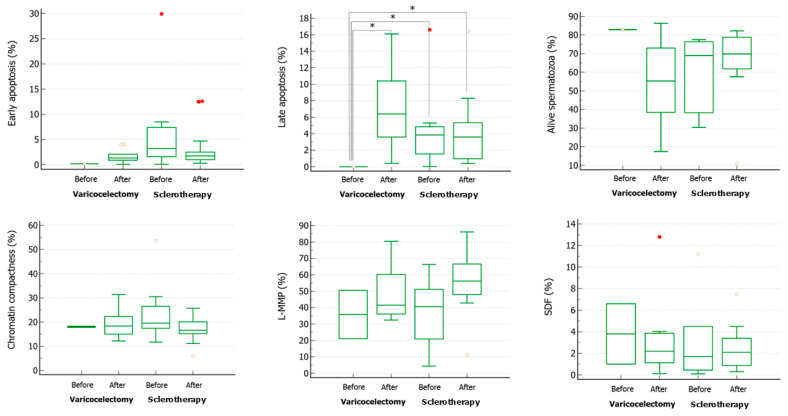
Bio-functional sperm parameters in patients with varicocele before and after undergoing varicocelectomy (Ivanissevich technique) or sclerotherapy. * *p* < 0.05 (one-way Analysis of variance).

Regarding the bio-functional sperm parameters, all the parameters analyzed did not change in a statistically significant manner compared to the values at enrollment in the two groups, except for the percentage of spermatozoa at late apoptosis, which increased significantly after varicocelectomy (Figure 4).

Finally, right and left testicular volume did not change after varicocele repair, neither in the varicocelectomy nor in the sclerotherapy group (right side: 14.0 ± 4.8 mL vs. 13.9 ± 4.3 mL, *p* > 0.05; left side: 15.4 ± 4.1 mL vs. 15.4 ± 5.0 mL, *p* > 0.05).

### 3.2. Patients with Oligoasthenozoospermia or Oligoasthenoteratozoospermia

When we limited the analysis to patients with oligoasthenozoospermia or oligoasthenoteratozoospermia only, we observed that the varicocelectomy group and the sclerotherapy group before repair differed only in total sperm motility values that were higher in the sclerotherapy group, and leukocyte concentration that was higher in the varicocelectomy group. In the post-operative period in the varicocelectomy group, there was a significant improvement in both semen volume and sperm concentration, while no significant effect was observed on the other conventional sperm and hormonal parameters. In the sclerotherapy group, on the other hand, there was a postoperative improvement not only in sperm concentration and total sperm count but also in progressive motility and leukocyte concentration, although for the latter parameter leukocytospermia (values > 1 million) was found in only three cases. The comparison between the varicocelectomy group and the sclerotherapy group did not reveal significant differences between the two groups in conventional sperm parameters and hormone levels post-treatment.

A comparison of bio-functional sperm parameters was not performed due to the limited amount of data available in this subgroup of patients (Table 2).

## 4. Discussion

The results of the present study showed that sclerotherapy significantly increased sperm concentration compared to pre-treatment values. Although the percentage of patients with oligoasthenozoospermia decreased in both groups, the reduction reached statistical significance only in the group undergoing varicocelectomy. Furthermore, sclerotherapy significantly increased sperm total motility compared to varicocelectomy. In contrast, the latter was associated with a statistically significant increase in serum LH levels and the percentage of spermatozoa in late apoptosis. This suggests that both Leydigian and tubular compartments may have suffered more from surgery. Limiting the analysis to patients with OA or OAT, a significant improvement in sperm count was found in both groups, but in the sclerotherapy group only there was also an improvement in total sperm count and progressive motility. However, the comparison of post-repair values between the varicocelectomy and sclerotherapy groups did not show significant differences, thus not highlighting the superiority of one treatment over the other in this subgroup of patients.

Other authors have compared surgical versus radiological treatments for varicocele repair (Table 3). One study compared four types of treatments, three surgical (retroperitoneal, inguinal, and sub-inguinal) and one radiological (percutaneous venous sclerotherapy) [24]. The study evaluated the efficacy in terms of sperm parameters improvement and the rates of recurrence and complications. All four therapeutic strategies increased sperm concentration after a follow-up of 6 and 12 months, but the sub-inguinal approach was the most effective followed by the inguinal, sclerotherapy, and retroperitoneal approaches. Moreover, the sub-inguinal approach also significantly increased sperm motility. Sperm morphology did not show any significant change in any of the groups after varicocele repair. The sub-inguinal treatment seemed to offer the lowest recurrence rate compared to the other techniques, although it was associated with a higher prevalence of hydrocele. Furthermore, due to its simplicity and the fact that it avoids the opening of the external oblique fascia, it represented the most plausible approach to clinical varicocele treatment [24].

Other authors have evaluated the success rate, morbidity, and costs of antegrade sclerotherapy compared to laparoscopic varicocelectomy. They have shown that antegrade sclerotherapy is the most successful treatment with a lower complication rate in patients with varicocele. Although both laparoscopy and sclerotherapy showed no major intraoperative complications, the three-month postoperative follow-up data showed a significantly higher number of complications after the laparoscopic procedure and, in particular, the development of hydrocele and the consequent need for hydrocelectomy. Local hematomas sometimes occur after sclerotherapy but without the need for specific treatment. Overall, the complication rates of laparoscopic varicocelectomy were approximately 2.5 times higher than those observed after sclerotherapy. In addition, the latter therapeutic approach had a disposable item cost approximately half lower than those used for laparoscopic surgery. Furthermore, local anesthesia use in sclerotherapy has reduced the number of preoperative tests and, consequently, the cost of the entire procedure [25].

A more recent study comparing the inguinal approach (Ivanissevich technique), scrotal sclerotherapy, and antegrade sub-inguinal sclerotherapy reported similar semen outcomes and pregnancy rates among patients undergoing varicocele repair with these three techniques. In the three groups, compared to baseline values, there was a significant increase in sperm count and total motility. At follow-up visits after 9 and 12 months, the increase in sperm count was greater in the group of patients treated with the Ivanissevich technique. Instead, at the six-month follow-up visit, sperm total motility increased in the groups treated with the Ivanissevich technique and sub-inguinal sclerotherapy. Progressive sperm motility and spermatozoa with normal morphology did not change significantly. Furthermore, the recurrence rate of antegrade sub-inguinal sclerotherapy was lower than in the other two types of treatment. Regarding complications, no case of hydrocele was found in patients undergoing sclerotherapy. This confirms that the approach mentioned above does not damage the lymphatic vessels as it simply dissects a vein which is then cannulated and injected [26].

The findings of the present study are in line with those of the latest Cochrane review. In detail, it confirms that varicocele repair positively affects the pregnancy rate in patients with abnormal sperm parameters and clinical varicocele. However, no evidence was found whether one treatment (surgical or radiological) can have a positive or negative effect on the live birth rate. Evidence on quality of life was lacking. Evidence of varicocele recurrence and adverse events (hydrocele formation, pain, epididymitis, hematoma, and suture granuloma) was also inconclusive. Therefore, a direct comparison of each treatment method did not allow us to achieve valid conclusions on the superiority of one therapeutic strategy over another regarding pregnancy, varicocele recurrence, or adverse events [19]. Therefore, the choice of procedure to be performed may depend on the cost and complication and post-operative recurrence rates. For example, laparoscopic surgical procedures were observed to be more expensive than other procedures. In contrast, there are no particular differences in costs between radiological and open surgical procedures. However, surgical procedures, especially retroperitoneal ones, as in our study, are generally associated with a higher complication rate ranging between 5 and 30% in retroperitoneal surgical procedures compared to 0–8% in anterograde embolization procedures. Furthermore, retroperitoneal techniques are associated with a higher risk of serious complications such as injury to lymphatic and arterial vessels compared to anterograde embolization and also a higher risk of developing post-operative hydrocele. Moreover, retroperitoneal surgical procedures would appear to be associated with a higher rate of recurrence [27]. However, it should be noted that in our study, we did not observe any differences in the recurrence rate, although the sclerotherapy group had a lower recurrence rate than the surgical group but not significantly so. Finally, no differences in the overall complication rate between the two procedures were found. These results are probably in part due to the low number of patients enrolled in the present study.

The European Association of Urology (EAU) strongly recommends treating infertile patients with clinical varicocele, abnormal sperm parameters, or other unknown causes of infertility in a couple whose female partner has good ovarian reserve to increase the fertility rate. The EAU also suggests advising patients with a high sperm SDF rate and otherwise unexplained infertility to undergo varicocelectomy or in case of failure after assisted reproductive techniques (ART), including recurrent pregnancy loss or failure of fertilization or implantation. Meta-analyses have compared different techniques for the treatment of varicocele. Overall, the microsurgical approach was associated with the lowest rates of complications and recurrences and the highest spontaneous pregnancy rate. However, it must be recognized that the microsurgical technique requires very specialized skills. In contrast, radiological sclerotherapy techniques are minimally invasive and can be performed under local anesthesia [28].

The American Urology Association/American Society for Reproductive Medicine (AUA/ASRM) guidelines suggest surgical varicocelectomy for patients who are seeking offspring and have palpable varicocele, infertility, and abnormal sperm parameters, but not patients with azoospermia. This is a moderate recommendation with a grade of evidence of B. However, the recommendation not to suggest varicocelectomy in patients with non-palpable varicoceles, identifiable only by imaging, is strong and has a grade C of evidence. Expert opinion agrees that no definitive evidence supports varicocele repair before ART in patients with clinical varicocele and non-obstructive azoospermia.

Overall, the results of the present study do not show a clear benefit of one technique over another. Our results, however, need to be taken with caution due to some limitations. First, this is a single-center study, enrolling a relatively low number of patients. It would be useful to have more data, preferably from different centers. It could also be useful to collect information after a longer follow-up period, although patients may be lost after longer follow-ups because they have achieved their goal of becoming fathers, have switched to other treatment options, or have abandoned their fatherhood plan. Furthermore, since the data refer only to the adult population of reproductive age, the results of this study cannot be applied to the adolescents with varicocele. The treatment of varicocele in pediatric and adolescent age is still a source of considerable debate that requires studies focused on this group of patients and this was not the aim of the present study [29,30]. Finally, the comparison with sclerotherapy was made only against retroperitoneal varicocelectomy, but not with other surgical techniques such as microsurgery or laparoscopic technique. Therefore, our results cannot be extended to all forms of surgical varicocele repair.

However, this study remains one of the few on the topic, which also considers sperm parameters among its outcomes and opens research to new considerations on the bio-functional sperm parameters.

## 5. Conclusions

Even today, varicocele remains a condition recognized as an etiological factor of male fertility. Treatment of a clinical varicocele can result in substantial improvements in sperm parameters and pregnancy rates. For good clinical practice, it is important to identify clinical varicoceles in infertile patients with abnormal sperm parameters. However, it is unclear what type of procedure clinicians should suggest to their patients, and it is useful to keep in mind that varicocele repair postpones ART treatment for at least 26 weeks. Therefore, it is necessary to identify the right patient to undergo varicocele repair and choose the most appropriate technique to use. For this reason, we need longer follow-ups and larger-scale, randomized, and controlled trials to update these results on the topic and resolve the issue. Finally, it could be useful to study other conditions that may influence the effects of varicocele on couple fertility such as the duration of the disease, the age, and the infertility factor of the female partner. It will probably be possible to carry out studies on different degrees of varicocele, in this way new results could be obtained that could guide towards the ideal treatment for the patient.

## Figures and Tables

**Table 1:** Characteristics of the patients at the enrollment.

	All Cohort (n = 94)	Varicocelectomy (n = 44)	Sclerotherapy (n = 50)	*p*-Value
Age (years)	29.0 (23.0–36.0)	29.5 ± 7.1	29.0 (23.0–35.0)	
Semen volume (mL)	3.1 (2.5–4.0)	3.3 ± 1.4	3.2 (2.5–4.2)	0.60
Sperm concentration (mil/mL)	10.0 (3.0–31.0)	12.0 (4.5–32.5)	8.0 (2.6–31.0)	0.44
Total sperm count (mil/ejaculate)	24.5 (8.8–112.0)	32.4 (16.7–118.5)	23.2 (6.0–110.0)	0.37
Progressive motility (%)	16.5 (6.0–30.0)	24.0 (8.8–34.3)	12.0 (6.0–28.5)	0.10
Total motility (%)	50.0 (30.0–63.0)	41.9 ± 24.8	48.3 ± 16.5	0.24
Normal forms (%)	4.0 (2.0–9.0)	6.9 ± 5.3	4.0 (2.0–8.0)	0.25
Spermatids (%)	2.0 (1.0–3.0)	2.0 (1.0–5.0)	2.0 (1.0–3.0)	0.83
Leukocytes (mil/mL)	0.4 (0.07–1.04)	0.85 (0.33–1.30)	0.20 (0.04–0.84)	0.01
Oligozoospermia (n,%)	8 (8.5)	5 (11.4)	3 (6.0)	0.87
Asthenozoospermia (n,%)	17 (18.1)	6 (13.6)	11 (22.0)	0.29
Teratozoospermia (n,%)	2 (2.0)	0 (0.0)	2 (4.0)	0.62
Oligoasthenozoospermia (n,%)	20 (21.3)	13 (29.5)	7 (14.0)	0.06
Oligoteratozoospermia (n, %)	1 (1.0)	0 (0.0)	1 (2.0)	0.93
Asthenoteratozoospermia (n, %)	6 (6.4)	2 (4.5)	4 (8.0)	0.49
Oligo-astheno-teratozoospermia (n, %)	25 (26.6)	7 (15.9)	18 (36.0)	0.03
Normozoospermia (n, %)	15 (16.0)	4 (9.1)	11 (22.0)	0.09
LH (mIU/mL)	3.8 (2.9–6.4)	3.9 ± 1.8	3.8 (3.0–5.8)	0.37
FSH (mIU/mL)	4.7 (2.8–6.4)	4.4 ± 2.2	4.4 (2.7–6.4)	0.51
TT (ng/dL)	5.4 (4.1–7.2)	5.0 (3.9–7.3)	5.7 (4.2–7.2)	0.76
Right TV (mL)	14.6 ± 4.7	12.5 ± 3.9	15.1 ± 4.8	0.17
Left TV (mL)	12.7 ± 4.2	11.3 (10.0–12.3)	12.9 ± 4.3	0.62
Early apoptosis (%)	2.4 (0.7–6.9)	0.2	3.2 (1.6–7.4)	-
Late apoptosis (%)	3.7 (1.0–4.6)	0	3.9 (1.6–4.9)	-
Alive (%)	61.6 ± 20.3	82.9	69.0 (38.3–74.4)	-
L-MMP (%)	36.8 ± 20.4	35.8 ± 20.8	37.0 ± 21.8	0.95
Chromatin compactness (%)	18.2 (17.8–22.5)	18.1 ± 0.4	19.6 (17.5–26.5)	0.60
SDF (%)	1.7 (0.6–4.5)	3.8 ± 4.0	1.7 (0.5–4.5)	0.60
Recidive rate (%)	29%	34%	24%	0.28
Overall complication rate (%)	6%	5%	8%	0.68
Hydrocele rate (%)	3%	2%	4%	0.99
After procedure pain rate (%)	2%	2%	2%	0.99

Abbreviations. FSH, follicle-stimulating hormone; LH, luteinizing hormone; L-MMP, low mitochondrial membrane potential; SDF, sperm DNA fragmentation; TT, total testosterone; TV, testicular volume. Values are expressed as mean ± standard deviation or as median and interquartile range in parentheses, according to the results of the Shapiro–Wilk test.

**Table 2:** Change in conventional sperm parameters and hormone values before and after varicocele repair within and between the two treatment groups.

	Before Sclero-Embolization	After Scleroembolization	*p*-Value	Befor Varicocelectomy	After Varicocelctomy	*p*-Value	Comparison Between Pre-Treatment Groups	Comparison Between Post-Treatment Groups
Volume (mL)	3.14 ± 1.5	2.96 ± 1.8	0.36	3.11 ± 1.27	3.52 ± 1.48	0.03	0.96	0.2
Concentration (mil/mL)	2.6 (1–5)	12 (2.5–21.75	0.0001	5.5 (1.63–7.5)	9.85 (4.8–21.8)	0.005	0.2	0.92
Total sperm count (mil/ejaculate)	6 (3–18.8)	27 (13.1–66)	0.0001	18.9 ± 19.3	28.6 ± 22.1	0.08	0.22	0.7
Progressive sperm motility (%)	6 (4.5–13)	15 (8–20.5)	0.005	13.5 (1–23)	12 (4–20)	0.62	0.7	0.6
Total sperm motility (%)	42.1 ± 16.8	50.8 ± 19.2	0.07	28.6 ± 22.1	37.3 ± 24.4	0.2	0.03	0.08
Normal forms (%)	3.82 ± 3.6	3.86 ± 2.86	0.95	6.8 ± 6.2	5.99 ± 4.2	0.6	0.3	0.07
Spermatids (%)	2 (1.75–3)	2 (1.75–2.5)	0.51	2 (0.15–5)	2 (0.1–2)	0.35	0.8	0.2
Leukocyte concentration (mil/mL)	0.27 ± 0.4	0.48 ± 0.53	0.02	0.61 (0.27–1.1)	0.55 (0.07–2.1)	0.95	0.006	0.3
Luteinizing hormone (IU/mL)	4.4 (3.9–6.7)	2.9 (1.96–8.8)	0.3	3.1 ± 1.1	4.2 ± 2.8	0.2	0.2	0.9
Follicle-stimulating hormone (IU/mL)	5.85 (2.9)	5.6 (3.3)	0.7	3.99 ± 2.35	5.06 ± 2.8	0.3	0.25	0.46
Total testosterone (ng/mL)	6.3 (4.3–7.2)	7 (5.8–25.9)	0.17	4.3 (3.9–10.4)	5.3 (4.2–6.6)	0.99	0.6	0.7

**Table 3:** Summary of the studies quoted in Section 4.

References	Participants	Treatment Types	Outcomes	Follow-Up and Study Duration	Conclusions
[24]	88 patients (23–39 years old) with clinical diagnosis of varicocele. Study excluded patients with subclinical varicocele or with other scrotal/urogenital diseases.	Participants underwent randomly to one of these approaches: 1. Surgical: -retroperitoneal; -inguinal; -sub-inguinal; 2. Radiological: -sclerotherapy.	1. Clinical occurrence of varicocele; 2. Hydrocele formation; 3. Sperm concentration; 4. Sperm motility; 5. Sperm morphology.	Study lasted from 1994 August to October 1996 and evaluated the outcomes after six months and twelve months post varicocele repair.	Sub-inguinal approach improved both sperm concentration and motility in the 6- and 12-month follow-up. Thanks to its simplicity and avoidance of opening the external oblique fascia, it represents the more plausible approach when treating clinical varicocele.
[25]	76 patients with mean age of 31 (16–45 years) and diagnosis of varicocele (clinically or by ultrasound scan). Study included patients who underwent treatment not only for subfertility but also inguinoscrotal pain and incidental finding with patient’s wish for treatment.	Patients randomly underwent to one of these approaches: 1. Antegrade scrotal sclerotherapy; 2. Laporoscopic varicocelectomy.	1. Therapeutic success (analyzing sperm concentration, motility and morphology) 2. Morbidity and complications 3. Costs.	The study evaluated patients from January 1997 to December 1999. Outcomes were analyzed at baseline and 3 months after varicocele repair with physical examination and ultrasonography.	Antegrade sclerotherapy is the less invasive treatment method for male varicocele with lower costs and better outcomes and should, therefore, be the preferred treatment method for male varicocele.
[26]	155 infertile patients with varicocele (diameter of the largest veins > 3 mm and time of regurge > 2 s during straining). Inclusion criteria were infertility for at least 1 year and subnormal semen.	According to patients’ preference, themselves underwent to one of these treatments: 1. Ivanissevich technique; 2. Scrotal antegrade sclerotherapy; 3. Sub-inguinal antegrade sclerotherapy.	1. Sperm count; 2. Total and progressive sperm motility; 3. Sperm morphology; 4. Pregnancy rates; 5. Baby take home rates; 6. Recurrence; 7. Complications.	Follow-up was performed every 3 months for one year by semen analysis, clinical and ultrasound evaluations.	Pregnancy rates and sperm parameters also showed no significant difference between groups. Only the increase in total sperm motility was less significant in scrotal antegrade sclerotherapy Instead the recurrence rate was lower for this technique. The sclerotherapy groups showed no hydrocele occurrence.

## Data Availability

The data presented in this study are available on request from the corresponding author.
